# Investigation on air quality of specific indoor environments—spa salons located in Gdynia, Poland

**DOI:** 10.1007/s11356-020-09860-4

**Published:** 2020-07-13

**Authors:** Klaudia Pytel, Renata Marcinkowska, Bożena Zabiegała

**Affiliations:** Department of Analytical Chemistry, Faculty of Chemistry, Gdańsk University of Technology, G. Narutowicza 11/12 Str., 80-233 Gdańsk, Poland

**Keywords:** Indoor air, Indoor air quality, Volatile organic compounds, Terpenes, Spa salons, Aromatherapy, Essential oils

## Abstract

Due to excessive application of essential oils and scented products in spa salons during aromatherapy and massage sessions, the elevated concentration of total volatile organic compounds (TVOCs), particularly terpenes, which are known as secondary organic aerosol (SOA) precursors, is expected there. This study was aimed at determination of VOCs with a particular regard to terpenes in air samples collected in selected spa salons located in Northern Poland. Active air sampling was conducted before and after treatments. Samples were analyzed with the use of thermal desorption gas chromatography coupled with flame-ionization detector (TD-GC-FID) and mass spectrometer (TD-GC-MS). Obtained results allowed to characterize chemical composition of indoor air of spa salons and also to relate the dependence between applied essential oil and indoor air chemical composition. It has been proved that (i) spa salons are characterized by TVOC concentrations exceeding recommended values of 300–400 μg m^−3^ in most of examined cases, reaching up to several thousand of micrograms per cubic meter, (ii) TVOC concentration is strictly related to salon characteristics and carried out treatments, (iii) terpenes constitute a significant part of TVOCs present in spa indoor air, from 22 up to 86%, (iv) most commonly investigated terpenes in the literature (d-limonene, α-pinene, camphene, and linalool) were also determined at the highest concentration levels in this study and (v) VOC chemical composition is strictly dependent on the type of applied essential oils. On the basis of obtained results, it may be stated that extensive application of essential oils rich in terpenes can significantly alter indoor air chemistry of spa salons, thereby influencing health and well-being of employees working there.

**Figure:**
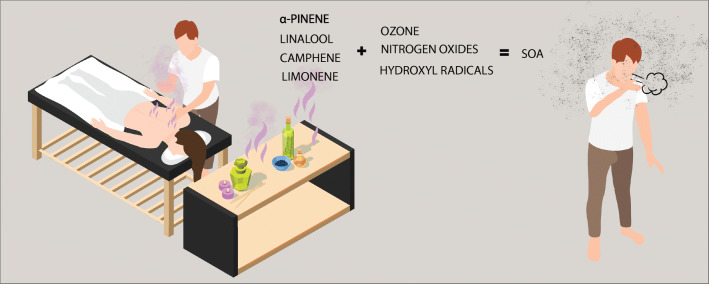
Graphical abstract

Graphical abstract

## Introduction

For several decades, indoor air quality has been a matter of interest of both scientists and politicians. This was triggered by the fact that people may spend up to even 90% of their time indoors, and therefore, chemical and physical transformations which occur indoors (sometimes referred as “indoor chemistry”) appeared as greatly important to human health and well-being (Samet 1993; Weschler and Carslaw 2018). Intensified interest in studying indoor air was triggered by proving that referring already gained knowledge of atmospheric air to indoor air may lead to inconsistencies, since mechanisms which govern indoor air chemistry are different from those characteristic to atmospheric air. Also, there are significant differences between these two environments in abundance of defined components and direct sunlight, temperature, and humidity fluctuations etc. (Weschler and Carslaw 2018; Abbatt and Chen 2020).

Indoor air chemistry is greatly affected by primary emission of volatile organic compounds (VOCs) from a wide range of sources present indoors, i.e., furnishing, building materials, everyday use products, human activities, and humans themselves (e.g., squalene, acetone) (Haghighat and De Bellis 1998; Klein et al. 2016; Lakey et al. 2017). Elevated VOC concentrations in indoor air pose a threat to human health, since a lot of VOCs (e.g., aldehydes, aromatics) (Liu et al. 2019) are documented to cause adverse health effects such as asthma and allergic reactions (Sofuoglu et al. 2011), as well as damage of the liver, kidneys, and nervous system. Moreover, some VOCs may exhibit a carcinogenic activity resulting in lung, brain, liver, blood, and kidney cancer (Rumchev et al. 2007).

Among VOCs, terpenes are a group of significant importance, since terpene-rich essential oils are components of used indoor furnishing, cleaning, fragrance, cosmetic, and cooking products. In addition, terpenes may be emitted also from natural sources such as plants and citrus fruits; however, it is believed that anthropogenic sources are those mostly responsible for elevated concentration of terpenes indoors (Wolkoff et al. 2000; Nazaroff and Weschler 2004; Tsigonia et al. 2010). Terpenes with one or more double bonding in their structure are highly reactive; hence, they instantly and easily undergo oxidation processes in indoor air such as ozonolysis (Weschler 2000; Atkinson and Arey 2003). Terpene ozonolysis initiates a number of chemical transformations, which lead to the formation of secondary organic aerosol (SOA) composition, especially in the first phase of rising, of nanosized (submicron) particles. These particles have been proved to pose a threat for human health (Rösch et al. 2017) since they are able to enter respiratory track and deposit along it by few mechanisms: diffusion, sedimentation, and impaction (Dockery et al. 1993; Spengler et al. 1996; Yeh et al. 1996; Pope and Dockery 2006). It has been proved that inhalation of SOA can cause some serious health effects such as inflammatory response in body tissues (Anderson et al. 2013), changes in lung cells, breath frequency decrease (Clausen et al. 2001; Sunil et al. 2007; Wolkoff et al. 2008, 2012), eye-blink frequency increase (Klenø and Wolkoff 2004; Nøjgaard et al. 2005), and even cancer (Pope and Dockery 2006).

There is a wide range of research carried out to determine VOC concentration with emphasis on terpenes in various indoor environments, e.g., homes (Król et al. 2014; Mickaël et al. 2014; Schlink et al. 2016), offices (Su et al. 2007; Dudzinska et al. 2012; Katsoyiannis et al. 2014), and schools (Larroque et al. 2006; Pegas et al. 2011; Markowicz and Larsson 2015). However, there is a limited number of research focusing on specific kind of indoor environments, where terpinene concentration is expected to be elevated, such as wineries (Sanjuán-Herráez et al. 2014), treatment plants (Gallego et al. 2012), elderly homes (Walgraeve et al. 2011), and beauty salons (Tsigonia et al. 2010).

Terpene-rich essential oils are widely applied as therapeutic agents during aromatherapy sessions known as alternative method of treatment. There are three main models of aromatherapy treatment: medical, where essential oils are most commonly delivered inside the body (oral, rectal, vaginal way); subtle aromatherapy, where essential oils are most commonly inhaled; and traditional aromatherapy, which is based on massage with essential oils (Dunning 2013). Aromatherapy is an area of growing interest, which becomes popular for example in psychiatry and oncology (da Silva Domingos and Braga 2014). Aromatherapy massages are very willingly used for relaxation; therefore, more and more people are inclined to undergo it (Grand View Research 2019).

Since terpenes are the main components of essential oils (Bakkali et al. 2008), a significant quantities of them are expected to be emitted into indoor air of spa salons during aromatherapy sessions. Since customers of spa salons usually spent there ca. 1 h, it can be assumed that exposition to elevated total volatile organic compounds (TVOCs) and terpene oxidation product concentration would not strongly affect clients’ health and well-being. Therefore, visiting spa for relaxation or taking physio- or aromatherapy session may actually be beneficial. However, employees of spa salons, by spending whole working day—usually 8 h—in spa indoor air may be exposed to elevated TVOCs and terpene oxidation product concentration, which may affect their health and well-being.

Worldwide, studies in this research area are focused most commonly on the determination of the composition either of the essential oil or the volatile fraction emitted from essential oil (or scented candles) that are usually applied in aromatherapy. Such studies are typically carried out with the application of a reaction chamber that mimics indoor air conditions, by direct GC analysis of essential oil solutions or with the use of SPME-based methodologies (Chiu et al. 2009; Huang et al. 2011; Cheng and Lai 2014; Ahn et al. 2015; Nematollahi et al. 2018). The aim of this research was to expand the knowledge and awareness related to temporary and permanent residence in indoor environments of specific types. Investigating indoor air quality in spa salons, where traditional aromatherapy session took place and, therefore, enhanced terpene evaporation into indoor air is expected, delivers valuable data useful for better understanding of indoor air chemistry. The main goal of this study was to determine the concentration of terpenes detected in indoor air samples in spa salons and to correlate carried out treatments and applied essential oils and cosmetic products with the chemical composition of indoor air. In the available literature, there are very few reported studies, in which sampling was carried out in real spa indoor environments, e.g., located in Taiwan (Hsu et al. 2012; Huang et al. 2012); however, a major emphasis has been put on SOA formation. To the best of our knowledge, this is the first study focused on VOC (with particular emphasis on terpenes) determination in such specific environments carried out in Poland.

## Materials and methods

### Sampling sites

**Fig. 1:**
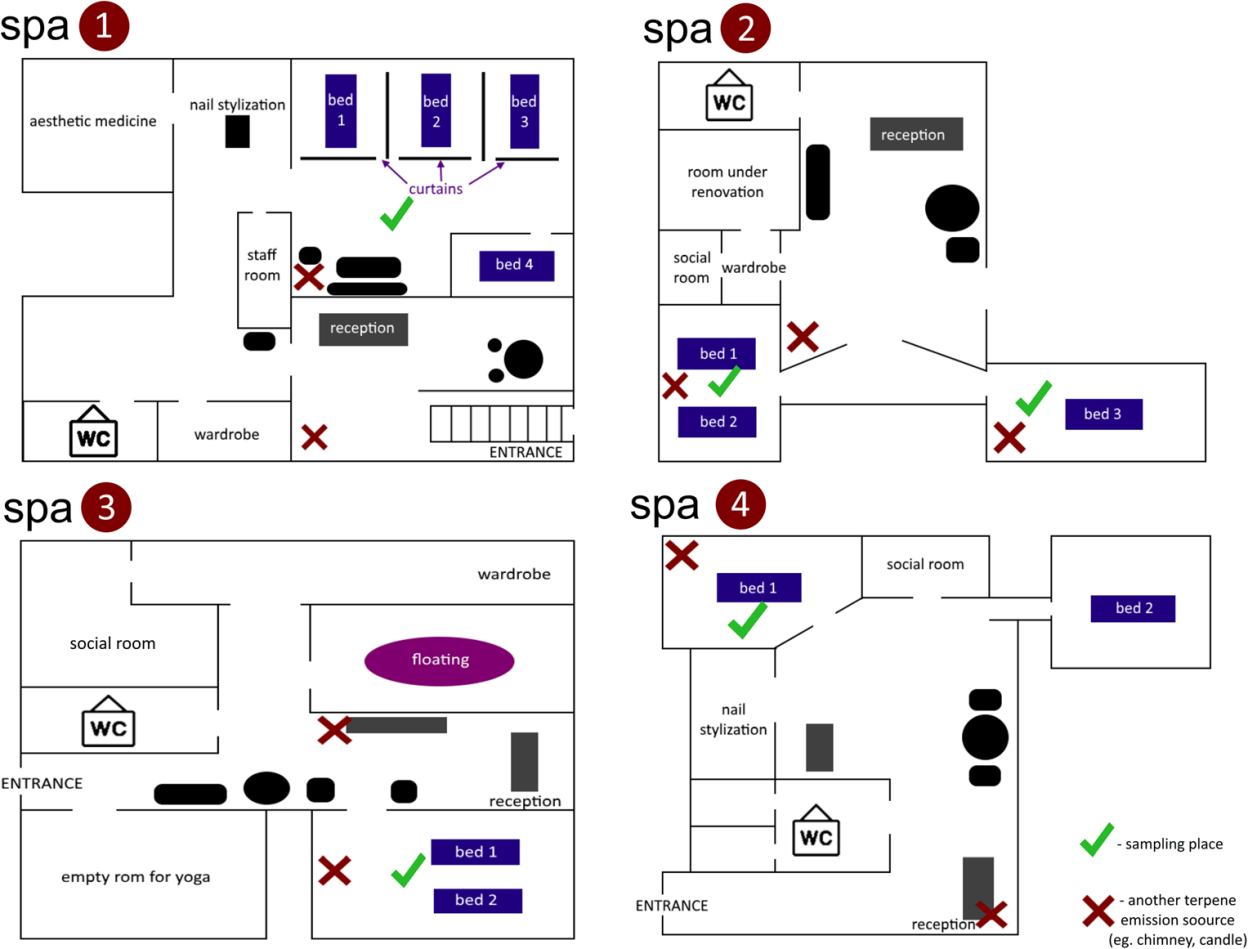
Schematic representation of room space arrangement of each of chosen spa salon

Four spa salons located in Gdynia (Poland) City center were chosen as sampling sites. Sampling campaign lasted from January 2019 to April 2019, and the sum of 60 samples was collected during campaign. Forty-four of them were analyzed with an application of thermal desorption and gas chromatography with flame-ionization detector (TD-GC-FID), and 16 of them were analyzed by thermal desorption and gas chromatography with mass spectrometry (TD-GC-MS). The brief characteristics of sampling sites together with schematic representation of their space arrangement (see Fig. 1) are presented below.

- Spa salon 1—typical spa salon, in which main treatments are aromatherapy massages with an application of large amounts of essential oils and body butters. Salons offers also esthetic medicine treatments and manicure, but those services are done in other rooms. Usually, clients are served during all day, which results in over a dozen of clients per day. For sampling, 4 and 13 samples were collected before and after treatment, respectively;
- Spa salon 2—massage center, in which relaxing aromatherapy massages and physiotherapy massages are carried out. This salon mostly serves regular customers, and visits are planned for whole day; therefore, over a dozen of clients is served daily. For sampling, 2 and 16 samples were collected before and after treatment, respectively;
- Spa salon 3—city spa which proposes few relaxing treatments: sensory deprivation, ganbanyouku, and ayurvedic massages. Massage room is equipped with two heated beds dedicated for ayurvedic massages. Spa 3 offers other treatments than massages; therefore, amount of daily performed massages is not regular and hard to define. It is common that number of massage clients rises due to periodic circumstances, e.g., Valentine’s day. For sampling, 5 and 14 samples were collected before and after treatment, respectively;
- Spa salon 4—health and beauty studio which is specialized in physiotherapy massages, which do not require application of fragrance compounds. Relaxing massages with essential oils are performed rarely. For sampling, 2 and 4 samples were collected before and after treatment, respectively. Limited number of samples is due to salon characteristics.

### Sampling

Air samples were collected before and after massage sessions (each session lasted 1 h). As sampling sorbent, Tenax TA® (Tenax TA 35/60®, 100 g, hydrophobic) was selected (Arrhenius and Engelbrektsson 2016; Petr and Soukupová 2017; Ramos et al. 2018; SIS 2019). Before sampling and after each analysis, sorbent tubes were conditioned for 6 h, at temperature of 300 °C in inert gas atmosphere using thermal desorption unit (Markes® Unity, Markes International, Great Britain). Conditioning was followed by a blank gas chromatography run (Agilent 7820A, Agilent Technologies Inc., USA with FID detector) to ensure no carry over effect. After conditioning, sorbent tubes were sealed with two-piece brass storage caps filled with one-piece PTFE ferrules (6 mm i.d.) and additionally closed in a screw cap glass vials for storage and transport. Samples were actively sampled via gas-tight syringe or automatic pump (constructed at Gdańsk University of Technology especially for the purpose of this study with constant air flow of 120 ml min^−1^). During sampling, 1 l or 2 l of air (depending on the carried out treatment) was actively passed through the sorbent tubes. During each sampling, there was one blank sorbent tube, which was sealed and placed near the sampling spot. Blank tube was maintained and handled the same way as sorbent tubes used for sampling. After sampling, sorbent tubes were sealed in the same way as described above. All samples were stored in a temperature not exceeding 20 °C, hidden from the sunlight, and were analyzed during max. of 48 h since sampling.

### Analytes of interest

The main goal of this work was to focus on determination of terpenes present in spa indoor air at high concentration levels; therefore, the main emphasis has been put on α-pinene, d-limonene, camphene, and linalool. Other VOC characteristics for each spa indoor environment, determined in this study qualitatively and quantitatively, are listed in Appendix Tables 2, 3, 4, 5, and 6. They represent such groups of chemical compounds as follows: alcohols, organic acids, esters, aldehydes, alkenes, alkanes, siloxanes, ketones, and terpene oxides.

### Chromatographic analysis and calibration

To carry out qualitative analysis, samples were subjected to thermal desorption (Markes® Unity, Markes International, Great Britain) and further to gas chromatography (Agilent 6890, Agilent Technologies Inc., USA) coupled with mass spectrometry (Agilent 5973 Mass Selective Detector, Agilent Technologies Inc., USA) (TD-GC-MS). Analytes were separated on DB-1 capillary column (Agilent Technologies; 60 m × 0.25 mm × 1 μm; 100% polydimethylsiloxane). To carry out quantitative analysis, samples were subjected to thermal desorption (Markes® Unity 2, Markes International, Great Britain) and further to gas chromatography (Agilent 7820A, Agilent Technologies Inc., USA) equipped with flame-ionization detector (TD-GC-FID). Analytes were separated on DB-1 capillary column (Agilent Technologies; 30 m × 0.32 mm × 5 μm; 100% polydimethylsiloxane). In both cases, chromatographic analysis was preceded with 10-min thermal desorption under 290 °C which was followed by transportation of desorbed analytes in the He stream (45 mL min^−1^) to the microtrap cooled down to 0 °C and subsequent heating of the microtrap to 300 °C for 5 min in order to release the analytes and direct them to chromatographic column. Temperature program of the GC-MS analysis was as follows: 50 °C, 10 °C/min to 280 °C. Temperature of ion source was 250 °C, while temperature of quadrupole was 150 °C. Temperature program of the GC-FID analysis was as follows: 40 °C for 10 min, 10 °C/min to 125 °C, 15 °C/min to 240 °C held for 5 min. Detector temperature was 250 °C.

For monoterpene concentration determination, five-point calibration curve was created using limonene ((R)-(+)-limonene standard, 97% purity, (Sigma-Aldrich, Poland) dissolved in methanol (gradient grade for liquid chromatography 99.9% purity, Merck) calibration solutions of following concentrations: 2.0; 4.0; 6.0; 8.0; and 10.0 ng μL^−1^. For each concentration 1 μl of calibration solution was introduced on previously conditioned sorbent tube, which was then flushed by a stream of nitrogen (99.999% purity) for 4 min. Afterwards, sorbent tube was sealed with two-piece brass storage caps, closed in a screw cap glass vials and quickly (up to 1 min) analyzed by TD-GC-FID. Each calibration solution was analyzed by TD-GC-FID in at least 3 repetitions. In order to obtain the best possible match, first two points of calibration curve were used to calculate LOD and LOQ values. For limonene LOD = 0.7 ng, LOQ = 2 ng which after recounting per 2-l air samples resulted in LOD = 0.35 μg m^−3^ (0.063 ppbv) and LOQ = 1 μg m^−3^ (0.18 ppbv). Percentage standard uncertainty associated with application of determined calibration relationship was calculated using Formula (1), (2), and (3). For limonene, percentage uncertainty is equal to 5.5%.

1 $$ u\left({x}_{\mathrm{pr}}\right)\%=\frac{u\left({x}_{\mathrm{pr}}\right)}{x_{\mathrm{pr}}} $$

where:

*x*_pr_—analyte content (half of calibration curve)

*u*(*x*_pr_)—standard uncertainty

2 $$ u\left({x}_{\mathrm{pr}}\right)=\frac{S_{\mathrm{x},\mathrm{y}}}{b}\times \sqrt{\frac{1}{p}+\frac{1}{n}+\frac{{\left({x}_{\mathrm{pr}}-\overline{x}\right)}^2}{Q_{\mathrm{x}\mathrm{x}}}} $$

where:

*S*_x, y_—standard deviation

*b*—slope

*p*—number of repetitions

*n*—number of repetitions for whole calibration curve

$$ \overline{x} $$—*x* mean value

3 $$ {Q}_{\mathrm{xx}}=\sum {\left(x-\overline{x}\right)}^2 $$

Concentration of other determined analytes (determined separately and as TVOCs) was calculated as toluene equivalents. Toluene (CHROMASOLV Plus, for HPLC, 99.9% purity, Honeywell) calibration solutions were prepared in the same way like limonene standard solutions. The five-point calibration curve was created using the following concentrations of toluene standard solutions: 2.0; 4.0; 6.0; 8.0; and 10.0 ng/μl. All of the calculations were performed similarly as in the case of limonene calibration. Therefore, the following values of metrological parameters were obtained: LOD = 1.9 ng, LOQ = 5.8 ng; for 2-l air samples MLOD = 0.95 μg m^−3^ (0.25 ppbv), MLOQ = 2.9 μg m^−3^ (0.77 ppbv). Percentage standard uncertainty was equal to 8.1%.

## Results and discussion

In 1992, ECA (European Collaborative Action) released Report 11 “Guidelines for Ventilation Requirements in Buildings,” according to which there are 4 comfort ranges of TVOCs indoors, proposing 300 μg m^−3^ as target guideline concentration for TVOCs indoors (European Collaborative Action 1992):

- < 200 μg m^−3^—comfort range
- 200–3000 μg m^−3^—multifactorial exposure range
- 3000–25,000 μg m^−3^—discomfort range
- > 25,000 μg m^−3^—toxic range

Unfortunately, only few countries have guidelines for indoor TVOC concentrations: Germany 300 μg m^−3^ (Seifert 1990), the USA 200 μg m^−3^ (USA-EPA 1996), Australia 500 μg m^−3^ (NHMRC 1993), Finland 200–600 μg m^−3^ (FISIAQ et al. 1955). In Poland, there is no regulation for TVOC concentrations; there are several but only for some specific VOCs. However, taking into consideration the values from other countries, one may state that TVOC concentration of 300–400 μg m^−3^ indicates that indoor air quality requires deeper investigation. Moreover, in Report 19, released by the ECA in 1997, it is mentioned that TVOC concentration above 25 mg m^−3^ increases the likelihood of sensory effects such as: dryness, sensory irritation, weak inflammatory irritation of eyes, nose, airways, and skin (ECA 1997).

The first step of this research was to determine, as recommended by the ECA (ECA 1997), as many volatile compounds as possible in collected air samples, at least those which are the most abundant. Qualitative analysis was done by TD-GC-MS. MS NIST 2.0 library was used to identify detected VOCs. On this basis, the list of most commonly occurring VOCs was created, which concerned compounds that were identified by NIST 2.0 library with probability higher than 70% (see Appendix Table 6).

**Fig. 2:**
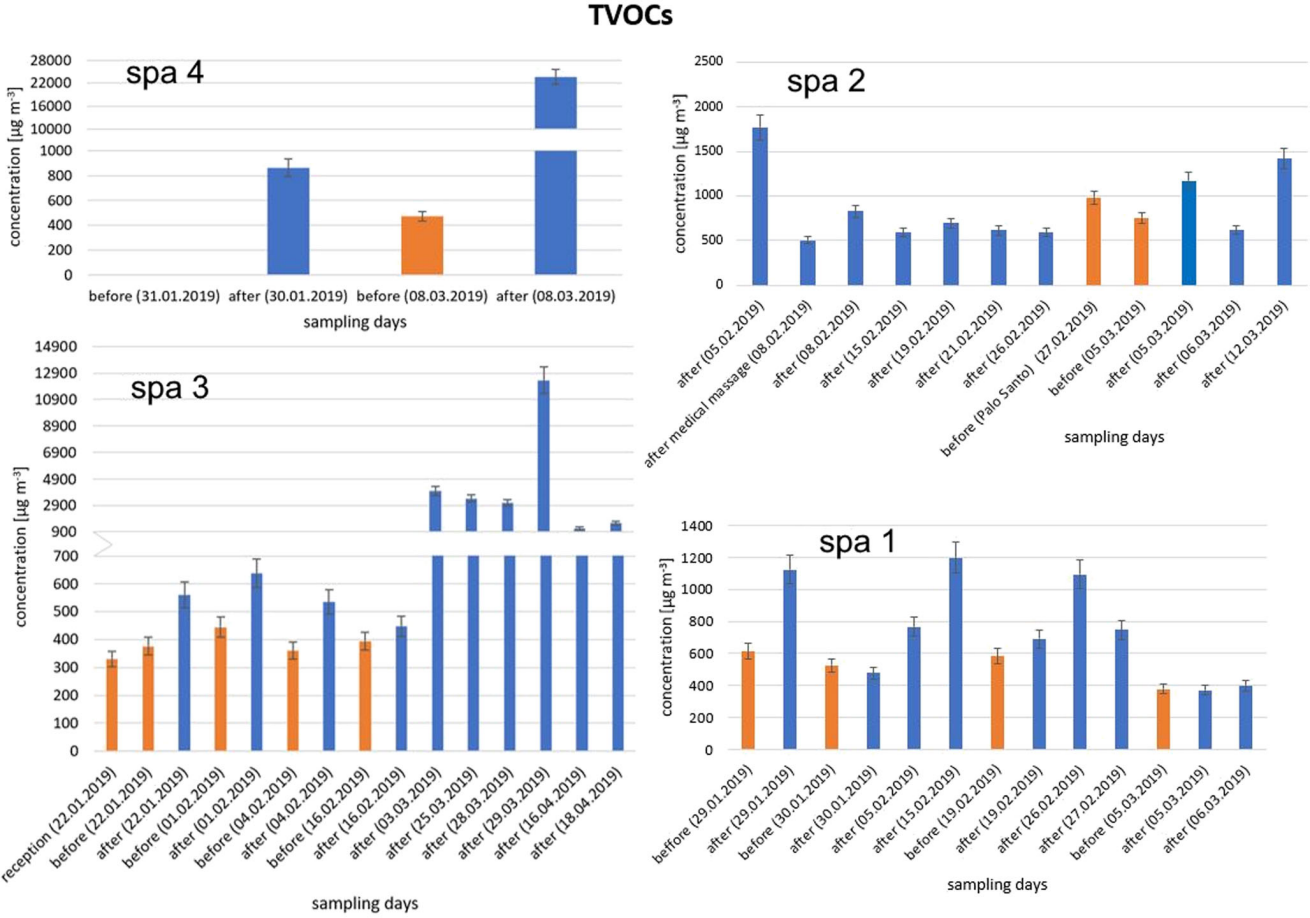
Variations in TVOC concentrations determined during sampling campaign in all investigated spa salons

TVOC concentration variations determined during all sampling days in all investigated spa salons are depicted in Fig. 2.

Determined instantaneous concentrations of TVOCs in all spa salons exceeded proposed target concentration of 300 μg m^−3^, even before the beginning of the massage. Most commonly, TVOC concentrations determined before treatment were lower than concentrations determined after the treatment, with two exceptions in spa 1 on the following sampling days: 30 January 2019 and 05 March 2019. High concentration of TVOCs measured before the beginning of treatments was probably related to high TVOC concentration in indoor air on the previous day. Most of the results are within the multifactorial exposure range. One exception is TVOC concentration measured in spa 4 after the massage—23,694 μg m^−3^, which was almost in the toxic range. However, because measured concentrations were instantaneous, it cannot be clearly stated whether they pose a serious threat to human health, as the effect depends on how long the exposition to high concentrations lasts. This particular spa salon (spa 4) is not specialized in very scented and relaxing massages. Usually, physiotherapeutic massages are carried out there. Moreover, the number of carried out treatments per day is not so high like in case of, e.g., spa 1, in which all three massage rooms are simultaneously occupied for most of the time.

Interesting fact is that in case of spa 2, concentration of TVOCs on 27 February 2019 was higher than on most of the other days when sampling was carried out after treatment. This was probably due to the smoldering of Palo Santo branch that day. According to the literature, Palo Santo (*Bursera graveolens*) essential oil is mostly composed of terpenes. Sotelo Mendez and co-workers (Sotelo Mendez et al. 2017) investigated the chemical composition of Palo Santo essential oil from Peru and determined that α-terpinene is a dominating component. It has been proved that the country of origin plays an important role in Palo Santo essential oil composition, since Fon-Fay et al. (2019) indicated that limonene constitutes 34.9% of the composition of *Bursera graveolens* essential oil from Ecuador, whereas according to the results obtained for essential oil from Peru (Sotelo Mendez et al. 2017), limonene constituted only 0.19% of its composition. In spa 2 on 27 February 2019, Palo Santo began to smolder approximately an hour before the official opening of the salon and more than one branch was used that day; therefore, it is highly probable that this activity was responsible for such high TVOC concentration measured, despite the fact that sampling was done before any massage started.

Generally observable trend was that TVOC concentration increased after each treatment in all spa salons. The greatest impact of the carried out treatment on the indoor air chemical composition was observable in the case of spa 4 on 08 March 2019. The TVOC concentration in sample collected after the massage was 55 times higher than that in the one collected before it started. During this treatment, a mixture of the following essential oils was applied: orange, lilac petals, Scots pine, and synthetic orange fragrance; whereas on the chimneys, there was lemongrass oil with wild rose and orange oils, which made this massage exceptionally aromatic. Taking into consideration the sharp increase of TVOC concentration after the massages with the application of essential oils, it may be stated that increased temperature inside the massage room, warm human skin and its large area contribute to intensified exposure of both workers and clients of spa salons to increased concentration of TVOCs, including terpenes (especially those of high volatility), in indoor air. This has been also proved by the results obtained by Huang et al. (2012) and Hsu et al. (2012) within their investigations on aromatherapy environments. In that studies, TVOC concentrations before massages was in the range 400–600 μg m^−3^ and 250–500 μg m^−3^ correspondingly, which is consistent with measurement in this research’s “initial” concentrations in the range 450–600 μg m^−3^. Huang et al. (2012) determined the highest TVOC concentration in 125–175 min of the measurement (during first aromatherapy session), and it reached 1200 μg/m^3^, while in Hsu et al. (2012) research, the highest noted TVOC concentration was equal to 3250 μg m^−3^ (for the details, see References). Determined in our study, TVOC concentration range of 600–1200 μg m^−3^ (with the exception of measured in spa 4 exceptionally high value of TVOC concentration) is close to the discussed above cases.

**Fig. 3:**
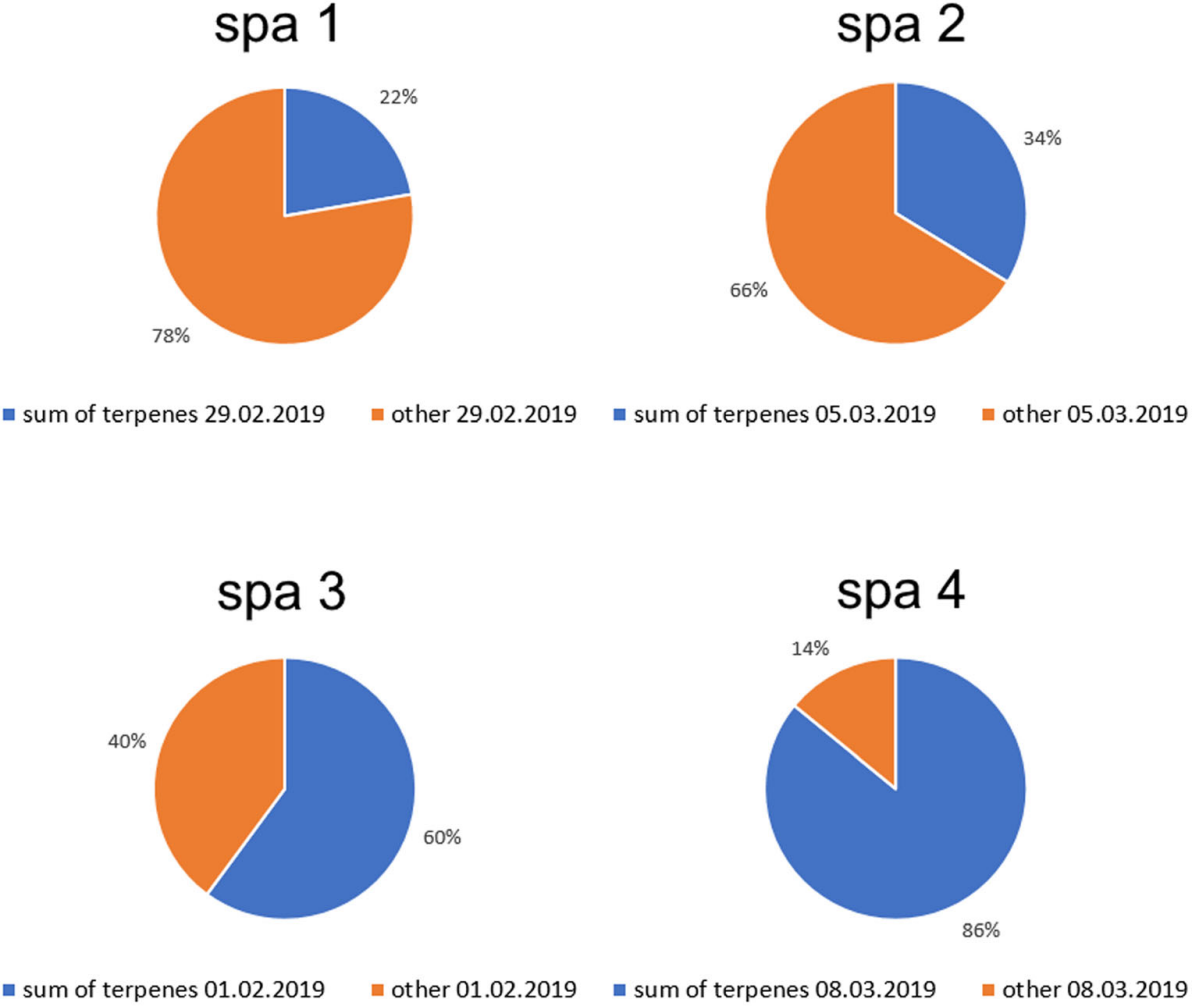
Percentage share of sum of terpenes and other VOCs in relation to TVOC content in air samples collected in investigated spa salons on exemplary sampling days

To get closer to the main purpose of this research, a percentage share of terpene concentration in relation to all determined TVOCs was calculated and presented in Fig. 3.

Applied during the treatment in spa 1, coconut and tea tree oils did not contribute significantly to percentage share of terpene concentration in relation to determined TVOCs. Relatively low (22%) content of terpenes in collected air sample was caused by the fact that coconut oil is not an essential oil and it is mainly composed of fatty acids (Marina et al. 2009); hence, the only source of terpenes in this case was tea tree oil heated on the chimney. In spa 2, a massage with an application of geranium and orchid oils resulted in elevated terpene percentage share in comparison to spa 1, which can be explained by the application of two essential oils during treatment. After the massage in spa 3, during which sesame and orange oils were applied, percentage content of terpenes in collected sample was equal to 60%, probably due to the fact that orange oil is mainly composed of limonene (77–95%) (Verzera et al. 2004; Tao et al. 2009). Application of large amounts of different essential oils during treatment in spa 4 (orange, lilac petals, Scots pine, lemongrass, wild rose) resulted in very large percentage share of terpenes in air sample composition equal to 86%. These results indicate that terpene content in spa indoor air depends on the type (chemical composition) and amount of applied oils and cosmetics during aromatherapy.

**Fig. 4:**
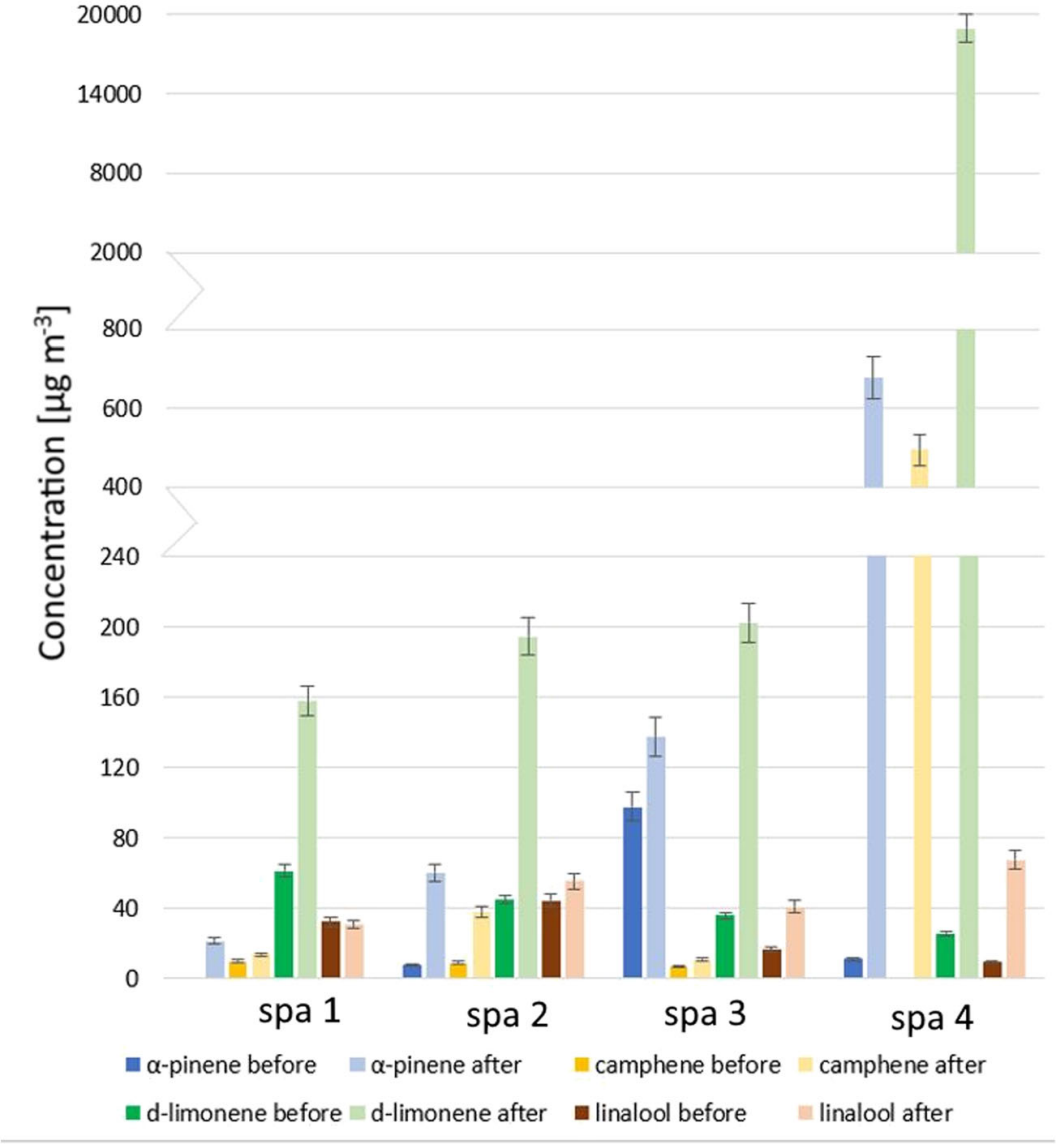
Variations of four representative terpenes determined before and after massage treatments in all investigated spa salons

According to the ECA, it is possible that specific VOCs may influence indoor air quality and may be solely responsible for or partially contribute to the development of health effects in greater extent than other VOCs. If such circumstances are suspected to occur, these specific VOCs (or one specific VOC) should be listed and determined separately (ECA 1997). Therefore, full list of 21 quantitatively determined chemical compounds is available in Appendix Tables 2, 3, 4, and 5, whereas four terpene compounds that were determined at highest concentration levels (α-pinene, limonene, camphene, and linalool) are discussed in detail below. According to the literature, these compounds are commonly investigated in terms of indoor air quality because of high abundance of their emission sources indoors. α-Pinene and limonene are the most commonly occurring terpenes in indoor environments. Camphene is also frequently determined terpene, but it is present in indoor air at concentrations lower than limonene and α-pinene (Tanaka-Kagawa et al. 2005; de Gennaro et al. 2013). Linalool is not so frequently determined in indoor air quality research as aforementioned terpenes, but it was proved that the application of essential oils is responsible for high emission of this compound (Su et al. 2007). Concentration variations of these selected compounds, determined by TD-GC-FID before and after massage treatment, are presented in Fig. 4. To date, terpene emission sources were the same as discussed above.

According to the results presented in Fig. 4, limonene was the most abundant monoterpene in all investigated spa salons and its background concentration (before treatment) varied from 25 to 60 μg m^−3^, which is similar to limonene concentration measured in studies on indoor air quality of shopping malls (Amodio et al. 2014) or homes (Villanueva et al. 2015). Concentrations of α-pinene, camphene, and linalool before treatments did not exceed 100 μg m^−3^. Concentrations of monoterpenes after massage treatment are visibly higher than before treatment, which indicates the presence of strong emission sources. Extremely high increase of limonene concentration up to 18,947 μg m^−3^ was probably caused by the use of complex essential oil mixture applied in huge amounts, which resulted also in increased TVOC concentration. Hsu et al. (2012) indicated in their research that limonene has very similar increasing concentration trend as TVOCs and its concentration in samples collected after massage was 16 to 60 times higher than in those collected before the treatment started. In our study, this range was even greater, since limonene concentration in indoor air after massage was from 2.5 to almost 740 times greater than before.

Since limonene is one of the most commonly occurring monoterpenes in indoor air and, as it was already mentioned, it was the most abundant monoterpene in all studied salons, we investigated particularly limonene concentration variations along all sampling days. Results of this investigation are presented in Table 1 as percentage share of limonene concentration in the sum of terpene concentrations. Such divergent results prove that each salon is a specific and characteristic environment with dynamically changing indoor air chemistry. All activities and applied cosmetic products have an influence on indoor environment of each of the salons; therefore, chemistry of spa salons cannot be unified and has to be specified for each of the salon separately.

**Table 1:** Concentration of limonene expressed as absolute value and percentage share in relation to a sum of terpene concentration measured within the research

	Concentration (ppbv) and percentage share (values in brackets [%])							
	Before treatment				After treatment			
	Min	Max	Arithmetic mean	Geometric mean	Min	Max	Arithmetic mean	Geometric mean
Spa 1	37.9 ± 2.1 (46.8)	146.1 ± 8.0 (71.1)	92.0 ± 5.1 (57.1)	74.4 ± 4.1 (56.2)	72.6 ± 4.0 (50.6)	108.5 ± 6.0 (73.9)	90.6 ± 5.0 (59.8)	88.7 ± 4.9 (59.3)
Spa 2	44.8 ± 2.5 (38.9)	141.4 ± 7.8 (52.8)	93.1 ± 5.1 (45.8)	79.6 ± 4.4 (45.3)	62.4 ± 3.4 (4.3)	105.5 ± 5.8 (59.5)	84.0 ± 4.7 (36)	81.2 ± 4.5 (29.4)
Spa 3	25.5 ± 1.4 (14.8)	35.9 ± 2.0 (20.9)	30.7 ± 1.7 (17.4)	30.2 ± 1.7 (17.3)	8.4 ± 0.5 (0.3)	1032.4 ± 56.8 (78)	520.4 ± 28.6 (47.5)	93.1 ± 5.1 (29.7)
Spa 4	< LOD (-)	25.6 ± 1.4 (38.8)	-	-	289.5 ± 15.9 (60.1)	18,950.8 ± 1042.3 (93.1)	9620.2 ± 529.1 (76.6)	2342.5 ± 128.8 (74.8)

**Fig. 5:**
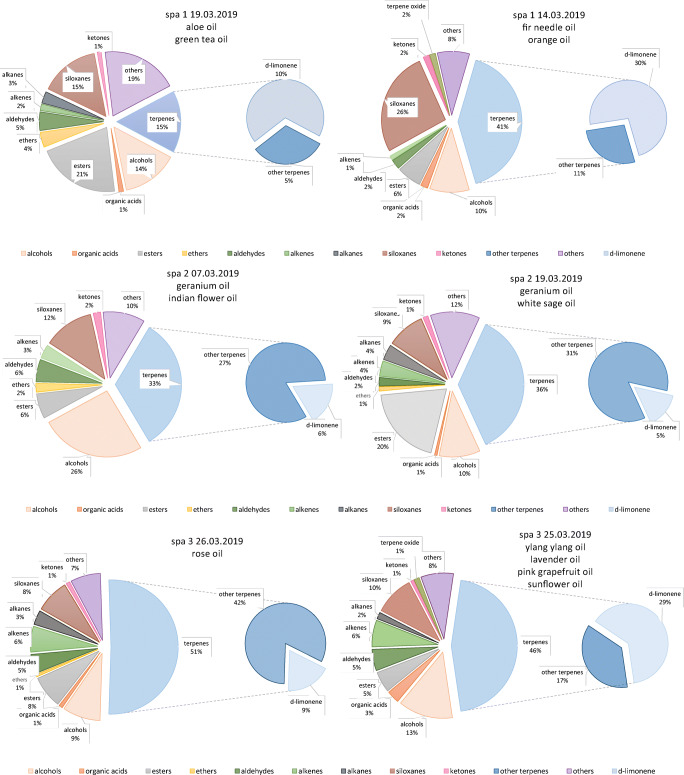
Percentage share of specific groups of compounds in air samples collected in spa salons with indication of applied essential oils during the treatments

In order to better describe the air quality of investigated salons, obtained qualitative results were combined to create charts showing percentage content of specific groups of chemical compounds in collected air samples. These results are presented in Fig. 5.

On the basis of collected data presented in Fig. 5, it may be stated that the type of the oil applied during the session highly influences the chemical composition of air inside the room where it took place. The presence of compounds of other than monoterpene groups of chemicals is probably related to the fact that commercially available essential oils (applied during investigated aromatherapy sessions) are rarely composed of 100% pure essential oil. It is very common that they contain additives such as alcohols, some other terpenes, and/or other fragrance compounds. However, the use of monoterpene-rich essential oils or cosmetic products with such essential oils, e.g., fir needle oil mainly composed of β-pinene (35–48%) (Régimbal and Collin 1994), orange oil mainly composed of limonene (77–95%) (Verzera et al. 2004; Tao et al. 2009), geranium oil rich in citronellol (37.5%) (Sharopov et al. 2014), or sage oil rich in 8-cineole (71.6%) (Borek et al. 2006), results in the dominant percentage share of terpenes in the investigated air samples. Geranium oil additionally contains a large fraction of alcohols in its composition (50–60%) (Babu and Kaul 2005), which also significantly influences collected air sample composition (see Fig. 5, spa 2 sampling day 07 March 2019). “Indian flower” is a commercial name of mixture of essential oils, which is unfortunately unavailable to check; therefore, it is impossible to speculate on its composition.

The high impact of the type (and composition) of essential oil during the treatment on the percentage content of limonene among all chemical compounds determined in air samples may be indicated with the example of spa 3 indoor air sample composition. Amounts of alcohols, organic acids, esters, ethers, aldehydes, alkenes, alkanes, siloxanes, ketones, and other compounds are very alike between two sampling days. A significant difference concerns limonene content. Rose oil applied on 26 March 2019 does not contain limonene; however, it contains significant amounts of other terpenes and scented terpene derivatives (geraniol 22%, nerol 10%, and citronellol 35%) (Ulusoy et al. 2009). Mixture of essential oils (ylang ylang, lavender, pink grapefruit oils) applied on 25 March 2019 changed limonene percentage share in sample chemical composition significantly. Ylang ylang and lavender oils contain trace amounts of limonene (Stashenko et al. 1996; Baratta et al. 1998; Hui et al. 2010); however, this terpene is a main component of pink grapefruit oil (88–91%) (Njoroge et al. 2005; Uysal et al. 2011); therefore, limonene was an abundant component of air sample collected that day.

Huang et al. (2012) and Hsu et al. (2012) in their studies determined that aromatherapy treatment causes submicron (< 100 nm) SOA particles growth at a level from 10,000 to 100,000 particles/cm^3^, indicating that high terpene concentration in such environments, even at low-level ozone concentration, produces large amounts of nanosized SOA. Additionally, terpene oxidation reactions in terpene-rich environments caused formation of formaldehyde up to 0.025 ppm, while background concentration was equal up to 0.005 ppm and I/O (indoor to outdoor ratio) indicated that formaldehyde emission sources were mainly indoors. Taking above into consideration together with obtained results within this study, it may be stated that indoor air quality in spa salons may pose a risk to human health and well-being.

## Summary and conclusions

This study, aimed at investigation of indoor air quality with particular emphasis on terpene presence in specific kind of environments such as spa salons, is, to the best of our knowledge, the first research of this type carried out in Poland. Results obtained within this study allowed to characterize indoor air quality and composition in selected spa salons. It has been proved that spa salons are characterized by TVOC concentrations exceeding recommended values of 300–400 μg m^−3^ and that TVOC concentration is strictly related to salon characteristics and carried out treatments. The evaluation of data gathered during this study indicates that terpenes constitute a significant part of TVOCs present in spa indoor air. Elevated terpene concentration is strongly related to the application of essential oils during treatments; however, constant emission sources such as candle chimneys filled with essential oils also contribute to increased terpene (and therefore TVOCs) concentration in indoor air. Indoor air quality in small spaces of spa salons can be easily altered by application of even small amounts of essential oils or cosmetics containing terpenes. It is known now that even small indoor concentration of ozone may trigger the reaction of SOA formation, as long as there is enough of second substrate—terpenes. Employees, by spending 8 h daily in such environment, may be at the risk of high exposure to harmful VOCs and terpene oxidation products, e.g., acetone, formaldehyde, and submicron SOA particles.

Indoor air monitoring in spa salons would be highly advised, as well as checking ventilation/air exchange performance. Further research in this area supported by toxicological studies would allow for obtaining results required for establishing and introducing of law regulations regarding maximum allowable concentrations of VOCs in these specific indoor environments.

## Appendix

**Table 2:** Determined concentrations of selected analytes in samples collected in SPA 1

	SPA 1 date												
	29.01.2019	29.01.2019	30.01.2019	30.01.2019	05.02.2019	15.02.2019	19.02.2019	19.02.2019	26.02.2019	27.02.2019	05.03.2019	05.03.2019	06.03.2019
	Before	After	Before	After	After	After	Before	After	After	After	Reception	After	After
Determined compounds	Concentration (ppbv)												
Isopropyl alcohol	20.64	n.d.	n.d.	20.92	n.d.	n.d.	n.d.	n.d.	n.d.	n.d.	n.d.	n.d.	n.d.
Acetic acid	24.67	n.d.	n.d.	n.d.	n.d.	n.d.	n.d.	n.d.	n.d.	n.d.	n.d.	n.d.	n.d.
Toluene	n.d.	0.79	< LOQ	0.90	n.d.	0.87	< LOQ	< LOQ	4.59	1.75	< LOQ	< LOQ	< LOQ
Cycli3siloxane	15.76	31.39	16.68	18.81	23.42	22.49	9.05	10.13	13.85	3.52	4.11	2.44	n.d.
2-Propanol	11.13	n.d.	16.30	8.17	23.60	32.09	n.d.	16.19	9.23	16.91	n.d.	n.d.	10.39
α-Pinene	< LOQ	3.79	1.95	1.05	3.06	4.48	1.68	3.62	2.81	2.55	2.38	< LOQ	1.74
Camphene	1.74	2.44	1.37	< LOQ	2.00	2.26	2.04	3.14	2.09	4.29	0.96	0.90	1.24
β-Pinene	< LOQ	1.66	1.02	< LOQ	1.25	2.39	1.05	1.76	8.09	< LOD	0.90	0.93	1.13
α-Phellandrene	1.18	1.36	1.11	1.26	1.72	2.68	1.07	2.96	3.15	6.50	1.19	n.d.	2.79
3-Carene	< LOQ	< LOQ	< LOQ	1.26	< LOQ	< LOQ	< LOQ	0.87	< LOD	< LOQ	n.d.	1.60	< LOQ
d-Limonene	11.02	28.37	7.11	3.91	11.89	13.06	26.28	34.75	30.16	< LOD	6.81	5.79	19.51
β-Phellandrene	n.d.	< LOQ	< LOQ	n.d.	< LOQ	0.92	< LOQ	< LOQ	2.18	< LOQ	< LOQ	1.79	< LOQ
Eucalyptol	0.85	1.82	1.02	< LOQ	1.64	1.34	2.98	7.82	6.06	n.d.	1.42	< LOQ	2.16
Linalool	5.10	4.88	3.13	0.80	5.16	6.31	4.65	7.05	4.58	7.86	2.77	< LOQ	2.39
Undecan	n.d.	n.d.	n.d.	n.d.	3.76	19.34	4.53	n.d.	5.05	n.d.	3.58	n.d.	3.73
Borneol	n.d.	n.d.	n.d.	n.d.	n.d.	8.13	n.d.	4.44	4.19	n.d.	2.28	n.d.	2.74
Cedrene/cryophyllene	3.52	4.96	2.44	n.d.	n.d.	n.d.	4.84	5.99	4.28	n.d.	2.30	n.d.	n.d.
Geraniol	< LOQ	n.d.	< LOQ	n.d.	0.97	< LOQ	< LOQ	< LOQ	n.d.	n.d.	< LOQ	n.d.	n.d.
Citronellal	n.d.	n.d.	n.d.	n.d.	2.46	2.70	2.31	5.71	8.08	1.82	2.27	n.d.	4.27
α-Amyl cinnamyl aldehyde	n.d.	n.d.	n.d.	n.d.	n.d.	n.d.	n.d.	n.d.	n.d.	2.99	n.d.	n.d.	n.d.
1,2-Dimethyl-3-nitrobenzene	n.d.	n.d.	n.d.	n.d.	n.d.	n.d.	n.d.	< LOQ	n.d.	n.d.	n.d.	< LOQ	n.d.

**Table 3:** Determined concentrations of selected analytes in samples collected in SPA 2

	SPA 2 date											
	05.02.2019	08.02.2019	08.02.2019	15.02.2019	19.02.2019	21.02.2019	26.02.2019	27.02.2019	05.03.2019	05.03.2019	06.03.2019	12.03.2019
	After	After	After	After	After	After	After	Before	Before	After	After	After
Determined compounds	Concentration (ppbv)											
Isopropyl alcohol	n.d.	29.26	21.26	28.44	7.36	60.90	56.81	n.d.	n.d.	n.d.	n.d.	n.d.
Acetic acid	n.d.	n.d.	n.d.	n.d.	n.d.	n.d.	n.d.	n.d.	n.d.	n.d.	n.d.	n.d.
Toluene	2.05	0.49	1.05	1.07	1.27	2.18	< LOQ	< LOQ	0.88		< LOQ	< LOQ
Cycli3siloxane	n.d.	< LOQ	< LOQ	0.80	0.89	0.78	n.d.	7.24	n.d.	0.95	< LOQ	1.56
2-Propanol	n.d.	n.d.	n.d.	n.d.	n.d.	3.69	n.d.	n.d.	n.d.	n.d.	n.d.	n.d.
α-Pinene	1.34	2.42	5.13	4.57	6.35	4.21	3.41	3.00	5.66	10.82	12.32	5.10
Camphene	1.34	1.62	3.18	1.69	2.48	0.87	2.17	2.52	1.59	6.87	2.48	3.48
β-Pinene	1.27	< LOQ	1.65	1.44	1.40	3.00	1.75	2.87	1.35	3.52	6.47	4.92
α-Phellandrene	0.80	0.99	1.59	1.25	0.96	2.75	< LOQ	3.34	1.35	1.28	2.17	< LOQ
3-Carene	6.26	1.70	1.97	1.96	2.09	3.84	< LOQ	1.67	2.72	2.58	3.50	7.01
d-Limonene	11.23	18.97	20.48	10.16	9.03	4.32	9.19	25.43	8.06	35.02	14.54	14.82
β-Phellandrene	237.98	6.21	3.92	5.47	2.74	7.21	n.d.	6.45	n.d.	10.74	31.88	9.63
Eucalyptol	1.07	2.42	7.55	2.10	5.16	< LOQ	2.16	3.44	5.21	5.40	4.53	12.92
Linalool	1.65	5.38	14.51	8.88	12.63	1.16	7.80	8.58	7.04	8.79	8.85	9.61
Undecan	n.d.	3.38		7.35	4.47	2.41	3.73	6.82	9.52	8.37	n.d.	10.66
Borneol	n.d.	1.51	4.49	1.59	3.66	n.d.	1.55	4.21	n.d.	n.d.	n.d.	n.d.
Cedrene/cryophyllene	n.d.	n.d.	2.04	1.04	1.75	n.d.	< LOQ	2.89	n.d.	n.d.	n.d.	n.d.
Lilial	n.d.	n.d.	n.d.	n.d.	3.60	n.d.	n.d.	n.d.	n.d.	n.d.	n.d.	n.d.
Geraniol	n.d.	1.03	3.29	0.97	1.11	n.d.	< LOQ	< LOQ	1.49	1.49	< LOQ	n.d.
1,2-Dimethyl-3-nitrobenzene	< LOQ	n.d.	n.d.	n.d.	n.d.	< LOQ	n.d.	n.d.	n.d.	n.d.	n.d.	n.d.
Citronellal	n.d.	n.d.	3.87	1.74	3.22	n.d.	n.d.	6.77	n.d.	n.d.	n.d.	n.d.

**Table 4:** Determined concentrations of selected analytes in samples collected in SPA 3

	SPA 3 date														
	22.01.2019	22.01.2019	22.01.2019	01.02.2019	01.02.2019	04.02.2019	04.02.2019	16.02.2019	16.02.2019	03.03.2019	25.03.2019	28.03.2019	29.03.2019	16.04.2019	18.04.2019
	Reception	Before	After	Before	After	Before	After	Before	After	After	After	After	After	After	After
Determined compounds	Concentration (ppbv)														
Isopropyl alcohol	2.56	31.43	8.00	21.47	23.94	35.15	28.93	21.61	n.d.	n.d.	n.d.	n.d.	n.d.	n.d.	n.d.
Toluene	1.44	2.21	2.23	2.28	2.62	1.72	2.12	2.87	1.82	n.d.	n.d.	5.67	n.d.	n.d.	5.15
α-Pinene	13.19	20.34	18.90	17.55	24.70	17.97	23.51	12.40	0.96	24.34	87.96	74.65	120.36	26.56	29.87
Camphene	1.34	1.12	1.79	1.17	1.89	1.18	1.56	0.95	< LOQ	7.20	15.51	10.52	46.55	9.86	5.85
β-Pinene	< LOQ	< LOQ	0.78	0.84	0.93	1.07	1.39	2.97	< LOQ	24.30	5.20	5.07	299.46	3.02	2.18
α-Phellandrene	< LOQ	< LOQ	2.02	< LOQ	< LOQ	< LOQ	1.98	0.83	0.82	n.d.	6.99	3.54	n.d.	4.12	1.46
3-Carene	4.44	4.90	4.82	4.90	5.03	5.38	5.74	6.54	3.88	389.20	20.11	16.97	114.74	8.73	6.97
d-Limonene	4.02	4.59	25.23	6.45	36.43	5.80	14.48	4.40	4.81	1.51	327.11	288.41	305.04	185.69	91.99
β-Phellandrene	< LOQ	< LOQ	n.d.	n.d.	n.d.	n.d.	n.d.	< LOQ	8.34	n.d.	n.d.	n.d.	71.55	n.d.	n.d.
Eucalyptol	< LOQ	< LOQ	0.94	< LOQ	< LOQ	< LOQ	< LOQ	< LOQ	n.d.	< LOQ	6.18	n.d.	n.d.	< LOQ	1.43
Linalool	2.43	1.79	0.93	2.60	6.49	1.63	5.70	1.14	93.00	2.98	30.69	4.97	117.50	23.85	19.10
Undecan	n.d.	n.d.	n.d.	n.d.	n.d.	n.d.	2.28	2.01	n.d.	n.d.	n.d.	n.d.	n.d.	n.d.	13.27
4-Methoxy benzyl alcohol	1.77	< LOQ	< LOQ	2.84	n.d.	< LOQ	n.d.	n.d.	n.d.	n.d.	n.d.	n.d.	n.d.	n.d.	n.d.
Geraniol	n.d.	n.d.	n.d.	n.d.	n.d.	n.d.	n.d.	< LOQ	n.d.	n.d.	n.d.	24.74	n.d.	n.d.	n.d.
Cireonellal	n.d.	n.d.	n.d.	n.d.	n.d.	n.d.	n.d.	< LOQ	n.d.	n.d.	1.2	n.d.	1.61	< LOD	< LOD

**Table 5:** Determined concentrations of selected analytes in samples collected in SPA 4

	SPA 4 date			
	30.01.2019	30.01.2019	06.02.2019	08.03.2019
Determined compounds	Before	After	Before	After
	Concentration (ppbv)			
Isopropyl alcohol	n.d.	n.d.	n.d.	3.25
Acetic acid	n.d.	n.d.	n.d.	n.d.
Toluene	n.d.	< LOD	0.82	n.d.
Cycli3siloxane	n.d.	n.d.	n.d.	1.25
2-Propanol	n.d.	n.d.	n.d.	n.d.
α-Pinene	n.d.	26.14	1.96	121.95
Camphene	n.d.	2.71	< LOQ	89.17
β-Pinene	n.d.	3.57	< LOQ	16.40
α-Phellandrene	n.d.	< LOQ	< LOQ	4.32
3-Carene	n.d.	2.16	< LOQ	17.84
d-Limonene	n.d.	52.08	4.61	3408.67
β-Phellandrene	n.d.	n.d.	n.d.	n.d.
Eucalyptol	n.d.	1.37	< LOQ	5.57
Linalool	n.d.	3.72	1.49	10.73
Undecan	n.d.	20.11	3.31	n.d.
Borneol	n.d.	n.d.	n.d.	2.12
Cedrene/cryophyllene	n.d.	n.d.	5.30	2.36
Lilial	n.d.	n.d.	n.d.	n.d.
Geraniol	n.d.	n.d.	< LOQ	2.68
α-Amyl cinnamyl aldehyde	n.d.	n.d.	< LOQ	n.d.

**Table 6:** VOCs identified by MS NIST 2.0 library with probability higher than 70%

SPA 1	SPA 2	SPA 3	SPA 4
Isopropyl alcohol	Isopropyl alcohol	Isopropyl alcohol	2-Methyl-1,3-butadiene
Acetic acid	1-Propanol	Acetic acid	Acetic acid
Ethyl acetate	1-Methoxy-2-propanol	Ethyl acetate	2-Butanone
Pentanal	Hexamethyl-cyclotrisiloxane	1-Butanol	Ethyl acetate
2-Heptanol	Hexanal	Pentanal	Toluene
Propylene glycol	4,5-Diethyl-octane	1,2-Dichloropropane	Hexamethyl-cyclotrisiloxane
Toluene	2-Methyl-octane	Propylene glycol	α-Thujene
Hexamethyl-cyclotrisiloxane	3-Methyl-octane	1-Pentanol	α-Pinene
Hexanal	Ethylbenzene	Toluene	Camphene
Butyl ester acetic acid	p-Xylene	Hexamethyl-cyclotrisiloxane	Cosmene
Ethyl ester 2-mehyl butanoic acid	Nonane	Hexanal	β-Phellandrene
p-Xylene	trans-1-Ethyl-4-methyl-cyclohexane	Butyl ester acetic acid	β-Pinene
Heptanal	2,4,6-Trimethyl-heptane	Ethylbenzene	3-Carene
1-Butoxy-2-propanol	3,5-Dimethyl-octane	p-Xylene	Limonene
α-Pinene	1-Ethyl-2-methyl-cyclohexane	Heptanal	Terpinene
Benzaldehyde	2,6-Dimethyl-octane	Bicyclo[4,2,0]octa-1,3,5-triene	o-Isopropenyltoluene
β-Pinene	3-Ethyl-2-methyl-heptane	Propanol	Decamethyl-cyclopentasiloxane
Hexyl ester acetic acid	Butyl-cyclopentane	α-Pinene	1-Methyl-4-(1-methylethenyl)benzene
1,1″-oxybis-2-Propanol	Propyl-cyclohexane	Benzaldehyde	ocimene
1-Methyl-4-(1-methylethyl)benzene	α-Pinene	β-Pinene	(E,Z)-2,6-Dimethyl-2,4,6-octatriene
Limonene	4-Methyl-nonane	2-Pentylfuran	Limonaketone
Benzyl alcohol	3-Ethyl-octane	Octanal	Naphthalene
Eucalyptol	1-Ethyl-3-methyl-benzene	1,2,4-Trimethylbenzene	1,7,7-Trimethyl-bicyclo[2,2,1]-hept-2-yl acetic acid ester
(E)-2-Octenal	1-Ethyl-2-methyl-benzene	p-Mentha-1,3,8- triene	
2,6-Dimethyl-7-octen-2-ol	1,2,3-Trimethyl-benzene	3-Carene	
Acetophenone	decane	2-Ethyl-1-hexanol	
Undecane	cis-1,4-Dimethyl-cyclohexane	1-Methoxy-3-methylbenzene	
Decamethyl-cyclopentasiloxane	4-Methyl-decane	Limonene	
3,7-Dimethyl-1,6-octadien-3-ol	1-Methyl-4-(1-methylethyl)-benzene	β-Phellandrene	
Phenylethyl alcohol	Limonene	Eucalyptol	
Phenylmethyl ester acetic acid	Eucalyptol	(E)-2-Octenal	
1,7,7-Trimethylbicyclo[2.2.1]heptan-2-one	2-Methyl-decane	2,6-Dimethyl-7-octen-2-ol	
Dodecane	1-Methyl-3-propyl-benzene	cis-Linaloloxide	
Gardenol	1-Ethyl-2,3-dimethyl-benzene	Acetophenone	
4-Carene	Undecane	Decamethyl-cyclopentasiloxane	
Naphthalene	Decamethyl-cyclopentasiloxane	(E,Z)-2,6-Dimethyl-2,4,6-octatriene	
2-Phenoxy-ethanol	3,7-Dimethyl-1,6-octadien-3-ol	Phenylethyl alcohol	
Dodecamethyl-cyclohexasiloxane	3,7-Dimethyl-decane	cis-Limonene oxide	
(S)-2-Methyl-5-(1-methylethenyl)-2-cyclohexen-1-one	Phenylethyl alcohol	trans-Limonene oxide	
2-(1,1-Dimethylethyl)-cyclohexanol	2-Methyl-undecane	Phenylmethyl ester	
Isobornyl acetate	Tritetracontane	1,7,7-Trimethylbicyclo[2.2.1]heptan-2-one	
2,2,4,4,6,8,8,-heptamethyl-nonane	1-Methyl-2-(1-methylethyl)-benzene	Tetradecane	
tetradecane	5-Methyl-2-(1-methylethyl)-cyclohexanone	α-Methylbenzyl acetate	
Nopyl acetate	1,7,7-Trimethylbicyclo[2.2.1]heptan-2-one	Cyclohexanol	
4-(2,6,6-Trimethyl-2-cyclohexen-1-yl)-3-penten-2-one	p-allyl-Anisole	p-Meth-1-en-8-ol	
4-(2,6,6-Trimethyl-2-cyclohexen-1-yl)-3-buten-2-one	p-Menth-1-en-8-ol	(Z)-3,7-Dimethyl-1,3,6-octatriene	
Lilial	(R)-3,7-Dimethyl-6-octen-1-ol	Dodecamethyl-cyclohexasiloxane	
Pentyl ester 2-hydroxy benzoic acid	(Z)-3,7-Dimethyl-2,6-octadienal	(E)-2-Decanal	
Diethyl phthalate	Dodecamethyl-cyclohexasiloxane	(S)-2-Methyl-5-(1-methylethenyl)-2-cyclohexen-1-one	
1,1″-Oxybis-octane	3-Methyl-6-(1-methylethyl)-2-cyclohexen-1-one	Tridecane	
Methyl ester 3-oxo-2-penthylmcyclopentaneacetic acid	Tridecane	2-(1,2-Dimethylethyl)-cyclohexanol	
Pentyl ester 2-hydroxy benzoic acid	(1S-endo)-1,7,7-Trimethylbicyclo[2,2,1]heptan-2-ol	Isobornyl acetate	
Isopropyl myristate	2-(1,1-Dimethylethyl)-cyclohexanol	2,2,4,4,6,8,8,-Heptamethyl-nonane	
1-Pentanol	Isobornyl acetate	tetradecamethyl-cycloheptasiloxane	
o-Xylene	4-tert-Bytulcyclohexyl acetate	α-Cedrene	
Camphene	3-Methyl-6-(1-methylidene)-cyclohexene	Cedr-9-ene	
Hexanoic acid	Butyl ester butanoic acid	Nopyl acetate	
3-Carene	Copaene	Tricyclocaryophyllene	
(E,Z)-2,6-Dimethyl-2,4,6-octatriene	4-(2,6,6-Trimethyl-2-cyclohexen-1-yl)-3-buten-2-one	Cedrene	
cis-Limonene oxide	Caryophyllene	[4,2,1,1(2,5)]Dec-3-en-9-ol	
trans-Limonene oxide	Pentadecane	Hexadecamethyl-cyclooctasiloxane	
trans-5-mrthyl-2-(1-methylethyl)-Cyclohexanone	4-(2,6,6-Trimethyl-2-cyclohexen-1-yl)-3-penten-2-one	Hexadecane	
Menthol	4-(2,6,6-Trimethyl-1-cyclohexen-1-yl)-3-buten-2-one	Ethane-1,1-diol dibutanoate	
1-(4-Methylphenyl)-ethanone	Lilial	Cedrol	
Terpineol	Hexadecane	Camphene	
Tetradecamehtyl-hexasiloxane	Diethyl phthalate	Decane	
n-Hexyl salicylate	Methyl ester 3-oxo-2-pentyl-cyclopentane acetic acid	1,2,4-Trimethyl-benzene	
	n-Hexyl salicylate	α-Phellandrene	
	Patchouli alcohol	1-Methyl-4-(1-methylehyl)benzene	
	Isopropyl myristate	Benzene	
	Styrene	Undecane	
	Camphene	Linalool	
	β-Pinene	Nonanal	
	4-Carene	(2R-cis)-5-Mehtyl-2-(1-methylethyl)-cyclohexanone	
	Limonene	p-Allyl-anisole	
	Eucalyptol	Terpineol	
	2,6-Dimethyl-7-octen-2-ol	3,7-Dimethyl-1,6-octadien-1-ol	
	Nonanal	3,7-Dimethyl-2,6-octadienal	
	Camphor	4-tert-Butycyclohexyl acetate	
	(2R-cis)-5-Mehtyl-2-(1-methylethyl)-cyclohexanone		
	Borneol		
	Acetic acid		
	2-Butanone		
	Ethyl acetate		
	Benzene		
	Propylene glycol		
	2-Mehtylpropyl ester acetic acid		
	Toluene		
	3-Methyl-1-butanol acetate		
	2,3-Dimethyl-bicyclo[2,1,]-hept-2-ene		
	2-Butoxy ethanol		
	1-Butoxy-2-propanol		
	Benzaldehyde		
	Octanal		
	α-Phellandrene		
	3-Carene		
	2-Methyl-6-methylene-2-octanol		
	Phenylmethyl ester acetic acid		
	1-(2-Butoxyethoxy)-ethanol		
	p-Methylbenzyl acetate		
	Naphthalene		
	β-Citronellal		
	Citral		
	1-Methyloctyl ester butanoic acid		
	Pentadecane		
